# Operative Dentistry

**Published:** 1887-01

**Authors:** J. A. Robinson

**Affiliations:** Jackson, Mich.


### Operative Dentistry.

BY DR. J. A. ROBINSON, JACKSON, MICH.

(Read before the Ohio State Dental Society, Toledo, October, 1886.]

To keep up with the improvements of the age for fifty years is no small task. It is a mark of genius to be able to make progress to the end of life; to overcome the hindrances that beset us and keep our feet from sticking to the clay of old age. The temptation of age is to rest, to work prescribed hours and draw pay; or not to work at all and be satisfied. We go back and lose power when we cease to become greater than our work, for to make progress at all, we must be greater than the labor we perform. When the work is greater than ourselves we invariably fall behind, become old fashioned and are superannuated. To make progress is the blossoming fruitage of what we have gained in information and culture; and in dentistry, in science in art, and in mechanics, and the art is only successful when carried out on scientific principles. As a profession we shall be what the aggregate of what each individual has become. If we fail it will be because there is not light and knowledge in the individuals; *but we shall not fail!*

The same humanity belongs to all, and effort of the mass will make each individual excel. Of course there will be different efforts, tastes, and different gifts; but effort of each individual will place us all on a higher level. If A succeeds better than B, it is, as a rule, because he has made greater effort. To keep pace with all the improvements in our own profession, we must be students, and read and understand every thing pertaining to our calling.

Operative dentistry means every operation upon the teeth and gums, and everything that pertains to the oral cavity. It means every appliance necessary to do the work of preserving the teeth, and some knowledge of therapeutics, chemistry, metallurgy, and mechanics, and the skillful use of tools. It means the improved methods of the old plans that were true in saving teeth. Ninetenths of the employment of the dentist is in filling teeth. The first requisite to good fillings is to be able to see what you are doing. So in molars and bicuspids the openings must be made free either by wedging or chiseling or the engine bur. It was a common practice in Boston fifty years ago among good operators to extract a molar or bicuspid tooth, if partially decayed, to save by filling the adjoining one, as the space would be sufficiently large to insert a good filling in the old fashioned way, with such instruments as could be obtained at that time; for every dentist was obliged to make his own instruments. Another essential is to prepare the cavity to receive the filling, and by this I do not mean so that the filling will not come out, but with such care as will make it easy and possible to hermetically seal it and make it impervious to all outside influences. What can I say to impress upon you the importance of properly preparing cavities? If you would spend one-half the time you spend in finishing your fillings in preparing the cavity, you would be great gainers in the end and do more good. Where there is chemical abrasion on buccal surfaces and a milky whiteness to the enamel, it is useless to try to preserve such teeth without that milky enamel is thoroughly removed.

In reading the descriptions of the Herbst method of filling teeth, I was pleased to learn that he stated that it was necessary to often examine the borders of the filling with a fine sharp point as the work progressed to see that the gold was absolutely in contact with the walls of the cavity, because the success of every filling depends on that word care. If the method be pellet, or ribbon, or Herbst, the success will depend largely upon such examinations of the borders with a fine instrument to see and know that your filling is absolutely tight against the walls, and any method will be a success that is done in that way.

Having been taught in the Harwood and Tucker school, it is easy and natural to follow the pellet plan with soft foil along the margins, and especially as some of our best young men still advocate their use. I think this method more nearly corresponds to the Herbst plan without the use of the engine, because he uses soft foil borders. But because we do this, we should not ignore the great advance that has been made by the combination of cohesive and of heavy foils. It is the skillful combination that makes success, and not insisting that either way is always and wholly right. Our successes in difficult operations are so much in advance of ancient dentistry that comparisons are inadmissible. All annealed cohesive foil should be as heavy as No. 6 and so on to No. 60, from the fact that the crystals have become so flattened in process of beating that it requires about 400 degrees of heat to raise them to the best welding point, and thin foils are liable to be burned at the edges in passing through the flame in process of annealing. Use finely serrated points near the finish, and lastly a smooth mallet burnisher. To be certain that there are no depressions in contour fillings that will disgust you, take a sharp straight file and file from point to cervical wall, as the carpenter would use his jointer to level a piece of wood, it will not destroy the welding properties in the depressions, but will show them that they may be filled before the final finish, if the file has been carried evenly across the face of the contour. How many times in large fillings we would give half the price of the work if we could finish out some little depression that annoys us. When a section of a tooth is to be replaced, if you have no screws, take a piece of thin gold plate of suitable width and the thickness of a separating file; bend it at right angles and place it in the hole drilled for the screw, leaving the trough toward that portion of the tooth that remains, and fill it tight into the hole and use it as you would a screw; the little grabs on either side of the gold will hold the filling even firmer than a screw and the gold trough is stiffer than the screw. Take strips of foil rolled on napkin-like cylinders, from one-half to three-quarters of an inch in length and weld one end on a line with the tooth, and returning weld the other end in the same line; then upset the end that comes below the point or cutting edge of the tooth, as a blacksmith would upset a piece of iron or steel for strength; lay these long strips lengthwise of the tooth and bind across the strips with heavy gold running to the edges of the cavity with gold as heavy as No. 60 and your contour will never come off. If the hand is too moist so it will rust your instruments, do not allow the gold to come in contact with it as it will largely destroy the cohesive quality. The best test is to rub the palms of the hands together, if they give a rustling sound, such as the farmer hears when he decides his hay is dry enough to be put into the barn, the hand will not injure the cohesive quality of gold. In finishing fillings, if you wish to do better than others, finish with strips; but if you wish to beat yourself, finish with disks; often done myself with very satisfactory results. Never use a flat-faced point near the border of enamel; foot pluggers should be conical on the face to prevent checking the enamel borders, similar to Butler's No. 16, only a longer foot, and the mallet burnisher should have the same form. The dentist is made strong by the combination of methods he includes, rather than by what he excludes, and his dentistry will be the same.

I have carefully looked through the whole catalogue of pluggers to find an universal one that is fit to weld cohesive to soft foil, or tin foil, or fibrous filling, and cannot find it. I will see if I can describe one: Take a No. 18 plugger, White's catalogue, and twist the point just half around like a cork-screw, so that it will be on a line with the shank, and have the twist begin three-fourths of an inch from the point, then sharpen the point to a stunt knife blade cutting edge; serrate with two serrations like No 18. This will leave three fine points with two serrations. Sharpen with a stone to an edge and it will unite everything. If you wish to reach undercuts have the twist shorter, but always observe the rule to twist half round and have the point to follow the shank to receive a direct blow from the mallet. By passing such an instrument as this round the filling near the margin of the cavity and spreading the gold against the borders, you can successfully stop all leak and at the same time prepare the surface for the heavy foil for the final finish. Of course use finer serrations and a mallet burnisher for the final finish. Cohesive gold will spread more easily over the soft foil base than a cohesive foil. Nine-tenths of the failures commence at the cervical walls in molars and bicuspid teeth, hence the necessity of great care in preparing the cavities. A spoon-shaped excavator will leave the cavity in better shape at this vulnerable point, and hand pressure is safer than mallet force. The matrix is essential to success. I use fibrous material in all dangerous places, believing that it is the best material known for the average operator. Fibrous filling is more easily finished, more compact, checks thermal changes, when it is finished it has antiseptic qualities, and if it turns black it is of no consequence if it only saves the teeth. It will not turn black unless it is overlaid with gold. When we can express with the fingers what is formed in the mind we shall succeed and not before. If there is a deep undercut at the cervical wall that impairs the integrity of the filling, there will be failure at this most vulnerable point. It is a good thing to wipe out deep-seated cavities with carbolic acid before filling, as it mitigates the pain in filling and also after the work is completed.

The successful operator in carrying out any system of filling teeth must carry out to the letter not only the plan laid down, but the instruments necessary to do the work. The Herbst method must have the point, the bulb and the engine if he expects success; so the filling with pellets must be done with graver pointed instruments to perfect the packing of soft foil. The best description I can give of the graver pointed plugger is a medium sized excavator with the point broken off one-third, and sharpened on all four sides, and on the end to a fine cutting edge on an oil stone. This instrument will carry the pellet before it and never pass through it, and having no serrates on the end will leave no pockets. In making pellets take a sheet of foil and cut it in halves and cut each half crosswise into four or five pieces. Crimp these pieces on the fore finger of the left hand or on a napkin with a knife, double them in the middle, fold in the edges and give it a slight roll between the thumb and finger. Before placing them in the cavity, anneal the butt end, and carry them to the walls of the cavity until they stand round the walls of the cavity like the fingers on the open hand, but always packing against the cervical walls first until the cavity is at least half full, and have each piece go to the bottom of the cavity and reach a little above the margin. By packing gold in this way and with this instrument you will keep your cavity as large at the bottom as it is at the top until the last piece or wedge of cohesive foil, and you will have a surface of annealed foil to receive a finishing of heavy cohesive foil. The Herbst method may supercede all other methods in time for the filling of teeth, but it will not become universal this year, so I have ventured to give an old plan to the young practitioners. The points of resemblance between the Herbst and the old are these: Herbst commences with a large cylinder; the Tucker plan with a large pellet. Herbst uses a German silver matrix soldered; we use a thin steel matrix tempered to a spring temper, or a matrix of wood. Both finish with cohesive foil; Herbst packs with rotary motion with smooth instruments and the engine; we use smooth sharp points and hand pressure. Both use soft foil margins. The folding of pellets belongs to Tucker, the graver point to myself. If after protracted operations you have soreness of the tooth and inflammation, chloroform is king of pain after filling, and in fact almost any degree of inflammation that precedes suppuration. Depletion of the gums with the fine point of a spear-shaped nerve instrument for sacrification, will immediately arrest all inflammation, and in cases of soreness I provide my patients with a small bottle of chloroform to take with them on leaving the office to bathe the gums after severe malletings. If you have exposed pulps and inflammation so the patient has toothache, treat with strong carbolic acid to paralyze the pulp and reduce the inflammation by depletion of one or two drops of blood, your success is almost certain if capped with oxyphosphates. The only danger in capping pulps is in the displacement of the cap. Two or three years ago I wrote about filling a tooth in sections to obviate that difficulty. First cut out the cap at the cervical wall and fill half full, and over one-half the cap with soft gold foil, textile foil, or tin to secure it in position, then remove the other part of the cap and fill in the usual manner, using great care not to crush the cap, as the little particles of the crushed cap will produce extravasation of blood that will be followed by inflammation and suppuration. If the work is properly done it will be successful and the pulp will remain alive.

In all remedies for cure of the disease known as pyorrhœa alveolaris the solution of the problem is to heal by first intention; the injured parts must be hermetically sealed to insure success. Whether the disease is produced by microbes or the excrementations that follow in their track is not positively known. If the disease is caused by acid excrementations, carbolized potash will neutralize the acid until it is harmless; and if it is caused by the microbes the carbolized potash is powerful enough to destroy their life, and it will close the pockets more effectually than any remedy I have known; and so I predicate my theory upon the hypothesis that having thoroughly removed all necrosed bone, if such a thing exists, by amputation, the lesion must be covered as in any accident to the human body or any wound you would heal by first intention. The carbolized potash applied on cotton fibres will extract the debris when the cotton is removed and cauterize the alveolar border and leaves a fresh wound to be healed from within, as all cure and all life must come from within. If the disease is caused by calcareous deposit, either in incrustation or loose particles combined with animal matter, and the general filth of the mouth to produce a pus sac that forces the margin of the gum from its normal position, there will be loose gums and a purple line along the border just below the festoon of the gum and it will stand out from the tooth; this must be removed with an instrument (a chisel) running toward the apex of the tooth before applying the remedy.

The carbolized potash is very efficacious in another form of disease known as sanguinary calculus caused by engorgement of the blood vessels along the border of the alveolar process, that produces a bright red line with small shoots or branches just below the border of the gum; but this will yield by persistent and frequent use of carbolized potash known as the Robinson Remedy. Before the word pyorrhœa had become generally received to designate this form of disease it was called struma or scrofula of the gums, and was treated with nitrate of silver, chloride of zinc, or creosote, on the point of a sharp stick, but it generally resulted in loss of the teeth; since we have more thoroughly understood the disease there are but very few cases that will not yield to treatment. In very obstinate cases where the secretions are ropy, stringy, and viscous, with dentine so sensitive you can hardly touch it, I pack the interstices and gums with prepared chalk every night for a week or ten days and success is certain. When carbolized potash is used as an obtunder to sensitive dentine or over an exposed pulp, it will produce slight pain; the potash turns the fatty portion of the pulp into soap and combining with the albuminous matter they both form an eschar that adheres to the surrounding border of the dentine and leaves the portion of the pulp as healed by first intention, and alive if the eschar is not broken in subsequent capping or filling of the tooth. There are sometimes cases (though sometimes means not often) of acute inflammation of the pulp that refuse to yield to conservative treatment, that must be destroyed and removed. In such cases apply the one-thousandth part of a grain of arsenic on fibres of cotton moistened with carbolic acid directly, if possible, to the pulp; after four or five days of rest remove the entire pulp from the pulp cavity and canals with a Donaldson nerve bristle, and enlarge the canal enough to have free access *and no more.* I use carbolic acid on cotton fibres in the pulp canal to remove soreness after the operation and fill the canals with cotton. Use absorbent cotton or common cotton as it is filled with oil and will not absorb moisture if any should remain to produce gas and inflammation. In the event of unexpected soreness after the operation of filling, drill a small hole to the pulp cavity and apply chloroform and you give immediate relief through the cotton, which cannot be done if the canal has been filled with oxyphosphates, gutta-percha, or metal. You will find among White's instruments a small, square handle, steel instrument, marked "Gate's patent," "special order," that is the only good, better, and best instrument I have found to put fillings in the canals after extracting the pulp. In cases where you have subsequent trouble and soreness, do not remove the filling but drill a small hole on a line with the root you wish to reach and treat with cotton fibres wet with chloroform or tincture of aconite and iodine, equal parts, until the soreness is removed, then fill the hole you have drilled and you will save all further trouble and save the tooth. Soreness of the tooth and toothache, though they may be combined, are not the same, and do not often proceed from the same cause, the one is inflammation of the pulp and the other inflammation of the surrounding membrane.

It may be maintained by some that it is not good surgery to place cotton at the mouth of a wound; but cotton fibres filled with carbolic acid or a fine rope that can be packed tightly at the foramen, form a complete eschar at the extreme end of the root like a cork in a bottle; and if the balance of the canal is left open to the pulp cavity it will do no mischief. I have had occasion to treat roots temporarily in this manner, and fill temporarily with Hill's stopping that have remained many months and when removed there was no odor or subsequent pain or trouble.

In this paper, (that is altogether too long,) I have only suggested some things that may help to leave "foot-prints in the sands of time," that will assist a brother on his way and contribute my mite to the meeting, and renew some old friendships with new expressions of love, without which, as St. Paul says, we are as "sounding brass and a tinkling cymbal."

